# The relationship between smartphone distraction and clinical decision-making among nurses

**DOI:** 10.1590/1806-9282.20260084

**Published:** 2026-08-28

**Authors:** Pınar Bekar, Seçil Aynur Çalık, Emine Efe

**Affiliations:** 1 Tokat Gaziosmanpaşa University, Erbaa Faculty of Health Sciences, Department of Child Health and Diseases Nursing – Erbaa/Tokat, Turkey.; 2 Burdur State Hospital, Coronary Intensive Care Unit – Burdur, Turkey.; 3 Akdeniz University, Faculty of Nursing, Department of Child Health Nursing – Antalya, Turkey.

**Keywords:** Clinical decision-making, Distraction, Nurses, Smartphone

## Abstract

**OBJECTIVE:**

The aim of this study was to examine the relationship between smartphone distraction and clinical decision-making among nurses.

**METHODS:**

This descriptive, cross-sectional study was conducted with 140 nurses working in a state hospital in a provincial center in the Mediterranean region of Turkey between 12 and 31 December 2024. Data were collected using the “Descriptive Information Form,” “Smartphone Distraction Scale,” and “Clinical Decision-Making in Nursing Scale.”

**RESULTS:**

A statistically significant weak negative correlation was observed between Smartphone Distraction Scale and Clinical Decision-Making in Nursing Scale scores (p<0.05). In addition, professional experience duration showed a weak positive relationship with Clinical Decision-Making in Nursing Scale scores (p<0.001).

**CONCLUSION:**

In this study, higher levels of smartphone distraction were associated with lower levels of clinical decision-making among nurses. In addition, longer professional experience was associated with higher levels of clinical decision-making.

## INTRODUCTION

Smartphone use is widespread globally and continues to increase in both developing and developed countries^1^. The Global System for Mobile Communications (GSM) Association^2^ reported that smartphones represented 80% of worldwide connections in 2024. Smartphones provide users with many opportunities like internet access, data storage, and social connectivity^3^.

Nurses can use their personal smartphones to access clinical information and support decision-making^1^. However, excessive smartphone use can reduce attention and increase distraction^4^. Smartphone-related distractions may significantly lower productivity and increase healthcare errors, creating serious challenges for patient safety and nurses’ clinical decision-making^5^. Clinical decision-making involves assessing patient needs, identifying potential health problems, and selecting appropriate interventions to ensure optimal patient care^6^. This process is fundamental to nursing practice and directly impacts patient health and well-being^7^. Nurses’ ability to make effective clinical decisions is essential for providing high-quality patient care and improving patient outcomes^6^. However, to the best of our knowledge, no study has examined the relationship between smartphone distraction and clinical decision-making among nurses. Accordingly, the aim of this study is to examine the relationship between smartphone distraction and clinical decision-making among nurses.

## METHODS

### Study design and participants

The research is descriptive and cross-sectional. The study population consisted of nurses working at a public hospital located in the city center of a province in the Mediterranean region of Türkiye (n=346). The required sample size was calculated as 55 participants using the G*Power statistical software based on regression analysis, with a significance level of 0.05, a power of 80%, and a moderate effect size (f^2^=0.15)^8^. Consequently, the study sample comprised 140 nurses who voluntarily agreed to participate in the study. According to the post hoc analysis, the effect size was found to be 0.029 (small), and this result suggests that the findings may be considered to have limited clinical significance in practice^9^.

### Data collection tools

#### Descriptive information form

The form included questions about the nurses’ descriptive characteristics^3,5^.

#### Smartphone Distraction Scale

Throuvala et al.^10^ developed a scale to assess smartphone-related attentional distraction, which has a 16-item, four-factor structure. The Turkish adaptation of the scale was carried out by Bilge et al.^11^ The subscales of the SDS are attention impulsiveness, online vigilance, multitasking, and emotion regulation. The scale is a five-point Likert-type scale, with total scores ranging from 16 to 80, where higher scores indicate greater levels of attentional distraction. The Cronbach’s alpha coefficients were 0.88 for attention impulsiveness, 0.80 for online vigilance, 0.76 for multitasking, and 0.76 for emotion regulation^11^. In the current study, Cronbach’s alpha was 0.81 for the total score, and 0.72, 0.77, 0.74, and 0.73 for the subscales, respectively, in the order mentioned above.

#### Clinical Decision-Making in Nursing Scale

The CDMNS, which was developed by Jenkins^12^. The validity and reliability study of the Turkish version of the scale was conducted by Durmaz-Edeer and Sarıkaya^13^. The CDMNS consists of 40 items and four subscales. The subscales are search for alternatives or options, canvassing of objectives and values, evaluation and reevaluation of consequences, and search for information and unbiased assimilation of new information^12,13^. The scale is a five-point Likert-type instrument. The total score of the scale ranges between 40 and 200. Higher scores on the scale indicate a higher perception of decision-making^12, 13, 14^. The Cronbach’s alpha coefficient was reported as 0.78 for the scale^13^. In this study, the Cronbach’s alpha coefficient for the scale was found to be 0.91 for the CDMNS.

### Data collection

Data were collected in person from nurses working at the state hospital where the study was conducted between December 12 and 31, 2024. Initially, the clinics were visited, and the nurses were informed about the study. Subsequently, verbal and written informed consent was obtained from the nurses who met the inclusion criteria and agreed to participate in the study, after which they were asked to complete the data collection instruments. The nurses completed the data collection forms in the nurses’ room. Completion of the forms took approximately 10–15 min.

### Ethics

Prior to data collection, ethical approval for the study was obtained from the Akdeniz University Medical Scientific Research Ethics Committee (decision no: TBAEK-468, dated 03.07.2024), and institutional permission was granted by the hospital where the study was conducted. Before data collection, all participants were informed about the purpose and procedures of the study, and verbal and written informed consent was obtained from all participants. No personally identifiable information was collected. Data were securely stored and used only for research purposes. The study was conducted in accordance with the Declaration of Helsinki.

### Data analysis

The results were analyzed using the Statistical Package for Social Sciences (SPSS) version 22.0 (SPSS Inc., Chicago, IL, USA). The normality of the data was assessed by calculating the skewness and kurtosis coefficients. Descriptive statistics (mean, standard deviation, frequency, percentage, minimum, and maximum), independent samples t-test, Pearson correlation analysis, simple linear regression analysis, and hierarchical regression analysis were used to analyze the data.

## RESULTS

The mean age of the nurses participating in the study was 36.09±8.03 years. Of the nurses, 92.1% were female, 82.1% were married, and 92.9% held a bachelor’s degree. It was found that 73.6% of the nurses worked rotating shifts, 66.4% were employed in clinics, and 89.3% were staff nurses. The mean duration of working in the profession of nursing was 14.30±8.26 years, and the mean total daily smartphone usage duration was 3.66±1.86 h.

The nurses’ mean SDS score was 36.75±7.96 for the total scale. Mean scores for the subscales were 8.63±2.55 for attention impulsiveness, 7.90±2.68 for online vigilance, 10.39±3.16 for multitasking, and 9.83±3.28 for emotion regulation.

The mean total score of the students on the CDMNS was 146.90±19.03. The mean scores for the subscales were as follows: search for alternatives or options, 37.51±5.45; canvassing of objectives and values, 37.18±5.43; evaluation and reevaluation of consequences, 37.31±5.85; and search for information and unbiased assimilation of new information, 34.91±5.43.

Mean CDMNS scores among nurses according to their descriptive characteristics were analyzed. It was found that the mean CDMNS scores of nurses differed significantly ­according to their work shifts. Nurses working the day shift had significantly higher mean CDMNS scores than those working rotating shifts (p<0.05). However, no statistically significant differences were found in the mean CDMNS scores of nurses according to gender, educational level, marital status, work unit, and job position (p>0.05).

The correlations between SDS and CDMNS scores, duration of nursing experience, and daily smartphone usage are presented in Table 1. There were statistically significant weak negative correlations between SDS and CDMNS scores (p<0.05). A weak negative correlation was found between the duration of working in the nursing profession and the SDS score (p<0.05), whereas a weak positive and significant correlation was found between the duration of working in the nursing profession and the CDMNS score (p<0.001).

**Table 1:** Correlations between Smartphone Distraction Scale and Clinical Decision-Making in Nursing Scale scores, duration of nursing experience, and daily smartphone usage.

		SDS	CDMNS	Duration of nursing experience	Duration of daily smartphone usage
SDS	r	–			
	p				
CDMNS	r	-0.170	–		
	p	0.044 (95%CI [-0.336, -0.001])			
Duration of nursing experience	rho	-0.272	0.317	–	
	p	0.001 (95%CI [-0.412, -0.103])	<0.001 (95%CI [0.140, 0.475])		
Duration of daily smartphone usage	rho	0.344	-0.218	-0.298	–
	p	<0.001 (95%CI [0.165, 0.495])	0.010 (95%CI [-0.384, -0.046])	<0.001 (95%CI [-0.440, -0.133])	

SDS: Smartphone Distraction Scale; CDMNS: Clinical Decision Making in Nursing Scale; CI: confidence interval.

Statistically significant weak positive correlations were observed between the duration of daily smartphone usage and SDS scores (p<0.001). Statistically significant weak negative correlations were identified between the duration of daily smartphone usage and the CDMNS scores (p<0.05; Table 1).

The model in which smartphone distraction (SD) level predicts Clinical Decision-Making in Nursing (CDMN) level was statistically significant (F=4.127, p<0.05). In this model, SD level explained 2.9% of the variance in CDMN level. A 1-unit increase in SD led to a 0.407-unit decrease in the CDMN level (p<0.05; Table 2).

**Table 2:** Results of simple linear regression analysis predicting Clinical Decision-Making in Nursing Scale from Smartphone Distraction Scale.

Independent variable	Unstandardized coefficients		Standardized coefficients	t	p	95%CI
	B	SE	β			
(Constant)	161.868	7.537		21.476	<0.001	[146.965, 176.771]
SDS total	-0.407	0.200	-0.170	-2.032	0.044	[-0.804, -0.011]

Notes: Durbin-Watson: 1.918; F: 4.127, p<0.05; R: 0.170; R2: 0.029; Adjusted R2: 0.022. CI: confidence interval; SE: standard error; **Β**: unstandardized beta coefficient; **β**: standardized beta coefficient; SDS: Smartphone Distraction Scale; CDMNS: Clinical Decision Making in Nursing Scale. Dependent variable = Clinical Decision Making in Nursing Scale.

A hierarchical regression analysis was conducted to examine the predictors of CDMNS scores, as presented in Table 3. In the first step, shift type and duration of professional experience were entered into the model as control variables. The first step of the model was statistically significant (F=13.911, p<0.001) and explained 15.7% of the variance. In this step, duration of professional experience was a significant predictor of CDMNS scores, whereas shift type was not a significant predictor.

**Table 3:** Results of hierarchical multiple regression.

	B	SE B	β	t	p	95%CI	F	Significance of the model	R^2^	Adjusted R^2^
Step 1										
(Constant)	133.255	3.000		44.413	<0.001	[127.322, 139.188]	13.911	p<0.001	0.169	0.157
Duration of nursing experience	0.848	0.182	0.368	4.669	<0.001	[0.489, 1.208]				
Work shift	5.728	3.391	0.133	1.689	0.093	[-0.978, 12.433]				
Step 2										
(Constant)	139.571	8.504		16.412	<0.001	[122.754, 156.388]	9.459	p<0.001	0.173	0.154
Duration of nursing experience	0.803	0.191	0.349	4.217	<0.001	[0.427, 1.180]				
Work shift	5.810	3.397	0.135	1.710	0.090	[-0.908, 12.528]				
SDS total	-0.155	0.195	-0.065	-0.794	0.429	[-0.541, 0.231]				

Notes: Durbin-Watson: 1.984. B: unstandardized coefficient; **β**: standardized coefficient; CI: confidence interval; SE: standard error; SDS: Smartphone Distraction Scale; CDMNS: Clinical Decision Making in Nursing Scale. Dependent variable = Clinical Decision Making in Nursing Scale.

In the second step, the SDS score was added to the model. The second step of the model was also statistically significant (F=9.459, p<0.001) and explained 15.4% of the variance. After adding SDS, duration of professional experience remained a significant predictor, whereas shift type and SDS scores were not significant predictors of CDMNS scores.

The findings showed that although a weak negative relationship was observed between SDS and CDMNS in the correlation and simple regression analyses, SDS did not emerge as an independent predictor of clinical decision-making when controlling for shift type and professional experience.

## DISCUSSION

This study examined the relationship between smartphone distraction and clinical decision-making among nurses.

In this study, the clinical decision-making level of nurses working the day shift was higher than that of nurses working in rotating shifts. In the literature, rotating shift work is common among nurses to ensure continuity of care. However, this system has been reported to be associated with numerous negative outcomes in terms of health and performance^15^. Additionally, Peng et al.^16^ found that fatigue caused by working the night shift impairs nurses’ decision-making and decision-making competence. The findings of our study are consistent with the existing literature.

Smartphone distraction may lead to significant challenges in nursing clinical decision-making processes^5^. In the present study, a weak negative relationship was found between smartphone distraction and clinical decision-making. However, smartphone distraction was not identified as an independent predictor of clinical decision-making. This finding suggests that smartphone distraction does not make a direct and independent contribution to the clinical decision-making process and that the observed relationship may be explained by other factors. Accordingly, these results suggest that reducing smartphone distraction alone may not substantially improve clinical decision-making.

The current study found a positive relationship between nurses’ duration in the nursing profession and their clinical decision-making levels. Additionally, professional experience was found to be a significant predictor of clinical decision-making. Consistent with this finding, studies reported that clinical decision-making was positively correlated with nurses’ work experience^6,17^. In addition, the literature emphasizes the important role of experience in nurses’ decision-making processes^18^. Therefore, effective clinical decision-making can be considered a skill that improves with experience.

In this study, it was found that as nurses’ duration in the nursing profession increases, their smartphone distraction levels decrease. Simone et al.^19^ found that nurses with fewer years of service reported greater distraction from non-work-related smartphone activities compared to those with more experience. Accordingly, it can be suggested that longer nursing experience may lead to increased awareness of distracting stimuli and more effective attentional control.

The current study revealed that as nurses’ daily smartphone usage duration increased, their clinical decision-making levels decreased. This finding is consistent with the literature, which suggests that excessive smartphone use may reduce attention span and increase distractibility, thereby limiting individuals’ ability to make appropriate decisions^4^. In this context, limiting smartphone use may support effective clinical decision-making processes.

The present study revealed that nurses’ daily smartphone usage duration is positively associated with smartphone distraction. This finding is consistent with a previous study^20^. In this context, strengthening smartphone-related time management and self-regulation skills may help mitigate smartphone distraction.

This study has several limitations. First, convenience sampling from a single hospital in Turkey limits the generalizability of the findings. Second, both the SDS and CDMNS are self-report measures completed at the same time point, increasing the risk of common method bias. In addition, no objective measure of smartphone use (e.g., screen-time records or direct observation) was collected, and the reported daily usage duration may have been affected by recall and social desirability biases. The CDMNS captures perceived decision-making ability, whereas the SDS reflects general smartphone-related distraction; therefore, observed associations may be influenced by subjective perceptions rather than actual clinical behavior. Finally, the weak correlations and limited explained variance suggest that the examined factors account for only a small proportion of the variance, and the findings should therefore be interpreted with caution. The use of observational or ecological momentary assessment methods may be recommended in future studies.

## CONCLUSION

In this study, smartphone distraction was negatively associated with clinical decision-making. However, smartphone distraction was not an independent predictor. Longer professional experience was associated with higher clinical decision-making. Increased smartphone use was linked to higher levels of smartphone distraction. In addition, nurses working day shifts demonstrated higher clinical decision-making than those working rotational shifts. These findings suggest that professional experience plays a more prominent role in clinical decision-making than smartphone-related factors. Strategies aimed at managing smartphone use in clinical settings and supporting less experienced nurses may help improve decision-making performance.

## Data Availability

The datasets generated and/or analyzed during the current study are available from the corresponding author upon reasonable request.
